# Medical and Philosophical News

**Published:** 1781-05

**Authors:** 


Mr. Henry Smeathman, an ingenious naturalift, intends foon to publifh an account of his travels travels in Africa and the Weſt Indies, in two volumes quarto. The continent of Africa and its productions are little known to Europeans, and have long been objects of curioſity to the lovers of natural hiſtory, ſome few of whom, it ſeems, ſuggeſted the plan of a voyage to that quarter of the globe, and enabled the author to embark with the neceſſary apparatus for making collections and obſervations in thoſe countries, where he reſided near eight years, promoting, as far as circumſtances would permit, the chief object of his travels.

Mr. Smeathman, however, did not confine his views merely to natural hiſtory, but likewiſe ſtudied the diſeaſes of Africa, which he means to deſcribe in his work, together with the different modes of treatment practiſed by the natives and by Europeans.

